# A curated multivariate approach to study efficacy and optimisation of a prototype vaccine against teladorsagiasis in sheep

**DOI:** 10.1007/s11259-023-10208-9

**Published:** 2023-09-14

**Authors:** Javier Palarea-Albaladejo, Tom N. McNeilly, Alasdair J. Nisbet

**Affiliations:** 1 Department of Computer Science, Applied Mathematics and Statistics, University of Girona, Girona, Spain; 2 Biomathematics and Statistics Scotland, JCMB, The King’s Buildings, Peter Guthrie Tait Road, Edinburgh, Scotland UK; 3 Moredun Research Institute, Pentlands Science Park, Bush Loan, Penicuik, Scotland UK

**Keywords:** Multivariate data analysis, Efficacy vaccine trials, Data reduction, Teladorsagiasis

## Abstract

This work discusses and demonstrates the novel use of multivariate analysis and data dimensionality reduction techniques to handle the variety and complexity of data generated in efficacy trials for the development of a prototype vaccine to protect sheep against the *Teladorsagia circumcincta* nematode. A curated collection of data dimension reduction and visualisation techniques, in conjunction with sensible statistical modelling and testing which explicitly model key features of the data, offers a synthetic view of the relationships between the multiple biological parameters measured. New biological insight is gained into the patterns and associations involving antigen-specific antibody levels, antibody avidity and parasitological parameters of efficacy that is not achievable by standard statistical practice in the field. This approach can therefore be used to guide vaccine refinement and simplification through identifying the most immunologically relevant antigens, and it can be analogously implemented for similar studies in other areas. To facilitate this, the associated data and computer codes written for the R open system for statistical computing are made freely available.

**Supplementary information:**

The online version contains supplementary material available at 10.1007/s11259-023-10208-9.

## Introduction

Statistical analysis in animal vaccine efficacy trials is traditionally focused on estimating and comparing responses induced by a prototype vaccine formulation against a placebo control group and other existing vaccine formulations. The aim is establishing a superior performance of the prototype in terms of reductions in pathogen burden and/or increases in animal productivity. Usually, the analysis includes data summaries and basic statistical testing for immunological parameters, such as pathogen-specific immunoglobulin G and A levels (IgG and IgA respectively); and of disease parameters, such as faecal worm egg counts (FEC) and worm burdens in the case of parasite vaccines (Bu et al. 2020). Ordinary pairwise correlations and simple linear regression are routinely used by researchers in the area whenever they are interested in unveiling associations between biological variables, e.g. between immunology and parasitology parameters. However, an overly simplistic statistical approach can provide only partial and limited information, or simply be misleading if key features of the data or the underlying biological processes are overlooked (Palarea-Albaladejo and McKendrick 2020). Recent vaccine efficacy studies in the area have introduced some statistical sophistication through the families of linear and generalised additive mixed models for longitudinal measurements of parasitology parameters (Cull et al. 2012; Nisbet et al. 2013, 2019) that allow explicit accounting for key aspects such as temporal correlation structure, nonlinear relationships with time, or heterogenous variability. Moreover, multivariate data analysis methods are an integral part of the statistical toolkit in areas such as chemometrics and bioinformatics (Varmuza and Filzmoser 2009; Ma and Dai 2011); however, to our experience they are hardly used in the context of vaccine efficacy studies. These methods are meant to analyse, visualise, and interpret complex datasets, including those with several variables up to hundreds or thousands as generated by modern high-throughput technologies. While some are extensions of univariate techniques such as the multivariate versions of analysis of variance and regression, others stand on their own right to accomplish tasks that are exclusive to multivariate problems and only feasible in practice thanks to the advent of scientific computing systems. Most multivariate techniques though are not inferential in nature and are instead primarily focused on data exploration and low-dimensional representation to facilitate visualisation and interpretation of patterns and relationships in the data.

In this work we present and demonstrate the novel application of a curated selection of multivariate methods that provide a global overview of the main drivers of vaccine-induced protection and contributes to identify associated immunological markers, which is key to inform vaccine refinement (Britton et al. 2020). The basics of the methods are introduced, stressing their purpose in the context of application and emphasising visualisation features. It is not the objective of this work to provide a comprehensive methodological review nor discussing technical properties in depth, but to show how a tailored multivariate approach contributes to make the most out of the complex data generated in vaccine efficacy trials and to enhance biological insights. We focus here on a vaccine development against the parasitic nematode *Teladorsagia circumcincta*, which is the principal cause of parasitic gastroenteritis in small ruminants in temperate regions worldwide. Using data gathered over five trials assessing a prototype recombinant cocktail vaccine (Nisbet et al. 2013, 2019), the relationships between antigen-specific antibody levels, antibody:antigen avidity measurements and parasitological parameters of efficacy are investigated and discussed.

## Materials and methods

### Overview of experimental setting and biological parameters

Five *T. circumcincta* vaccine trials were conducted between 2010 and 2017 following identical design, protocols and procedures (see Supplementary Information, Section S1, outlining the general immunisation and sampling regime; further details in Nisbet et al. 2013, 2019). The trials were all performed on the same farm under comparable conditions and following the principles of randomised controlled trials. Texel crossbred lambs were reared under comparable conditions and randomly allocated to balanced treatment groups to be independently individually applied either the vaccine or placebo preparation by injection. Ages and characteristics of the animals were homogeneous within each trial, with ages varying between 3 and 7 months across trials. In Trial 2, two groups of vaccinated and control animals were formed and euthanised either 7 weeks or 11 weeks after the start of the infection (denoted Trials 2a and 2b respectively). In Trial 4, two different ages of lambs were used: 3-month-old and 6-month-old lamb (denoted Trials 4a and 4b respectively).

A recombinant protein eight-antigen prototype vaccine formula was used in all trials, comprising the 8 antigens Tci-APY-1, Tci-ASP-1, Tci-MIF-1, Tci-TGH-2, Tci-SAA-1, Tci-CF-1, Tci-ES20 and Tci-MEP-1 and formulated in the adjuvant Quil A to boost the immune response. Lambs in all control groups were immunised with Quil A adjuvant only. Lambs were immunised on three occasions three weeks apart and at the third immunisation, each lamb was challenged orally with 2,000 *T. circumcincta* infective larvae, three times per week for 4 weeks.

The following subsections “Immunology parameters” and “Parasitology parameters” describe the immunological and parasitological response parameters that were identically measured in individual lambs in each one of the trials. Sex (female, male) and weight gain (in Kg) were recorded as well, although note that weight gain was only available for vaccinated animals in Trials 1, 2a, 2b and 5.

#### Immunology parameters

Antigen-specific antibody levels (IgG and IgA) in serum and in mucus were measured by ELISA as described in Nisbet et al. (2013). Serum antibody and avidity levels were measured 2 weeks after the final vaccination, when antigen-specific antibody levels were at their peak. Mucus antibodies were harvested at post mortem, approximately 60 days after the final vaccination. Briefly, plates (Greiner Bio-one, high binding) were coated with each recombinant antigen (50 µl per well at a concentration of 1 µg/ml for IgG and 5 µg/ml for IgA). Serum or mucus was diluted 1:5000 for IgG and 1:10 for IgA in Tris Buffered Saline (20 mM Tris, 150 mM NaCl, pH 7.4) containing 0.1% Tween® 20 (TBST). The secondary antibody used for IgG detection was mouse monoclonal anti goat/sheep IgG-HRP conjugate (Clone GT-34, Sigma A9452), used at 1:2000 and, for IgA, mouse monoclonal anti ovine/bovine IgA, used at 1:20,000 (Clone K84,2F9, Bio-rad AbD Serotec, MCA628GA). The tertiary antibody for detection of IgA was rabbit anti-mouse IgG-HRP conjugate (Dako P0260) used at 1:1000. Antibody avidity was determined in sera from each animal using an additional potassium thiocyanate elution step as described in Nisbet et al. (2019). Briefly, after antigen/sera incubation, microtiter plates were washed six times in PBST (137 mM NaCl, 2.7 mM KCl, 8.1 mM Na_2_HPO_4_, 1.5 mM KH_2_PO_4_, 0.1% v/v Tween20, pH7.4), then duplicate wells for each animal incubated with 0, 0.25, 0.5, 1, 2, 3, 4 and 5 M potassium thiocyanate solution for 10 min at room temperature. Plates were washed a further six times with PBST, then residual antibody binding detected according to the standard ELISA protocol described above. The absorbance readings from wells with no potassium thiocyanate represented total antibody binding and absorbance readings in the presence of increasing concentrations of potassium thiocyanate were converted to a percentage of total binding. Data were fitted to a graph of log_10_ molar concentration of potassium thiocyanate *versus* normalised response by non-linear regression to estimate half maximal inhibitory concentration (IC_50_) values.

- *IgG avidity index*. This measures the strength of the binding between each of the antigens and the antigen-specific IgG antibodies in vaccinated animals. The induction of higher antibody avidity may be indicative of higher vaccine efficacy.
- *Antigen-specific IgG and IgA in serum and in abomasal mucus*. This measures the levels of antigen-specific total IgG and IgA antibody response (values normalised across all trials by using the same plate positive control).

The avidity dataset included a small fraction of missing values in Tci-APY-1 (4%) and Tci-CF-1 (1.33%). They were replaced by predictive mean matching imputation (Schenker and Taylor 1996) based on the information provided by the observed data. Note that for Trial 4 IgG antibody responses in serum were available only for vaccinated animals and it was then removed from the analysis of antibody responses to facilitate comparability across trials, treatment groups and datasets. The immunology parameters generally showed right-skewed distributions and were log-transformed to better accommodate symmetry and linearity assumptions.

#### Parasitology parameters

Faecal egg counts (FEC) were measured three times per week from 14 days after the start of the parasite challenge until the end of the experiments. A modification of the salt flotation faecal worm egg count with a sensitivity of up to one egg per gram was used (Jackson 1974). Abomasal nematode burdens were classified and enumerated following standard techniques.

- *Percentage reduction in cumulative faecal egg counts (% cFEC reduction)*. Cumulative FEC (cFEC) for each animal in each trial was calculated using the trapezoidal method. They were normalised to facilitate comparability across trials by using $\lbrack1-\left(\text{cFEC}/-{\overline{\text{cFECc}}}\right)\rbrack \cdot 100$; that is, as the reduction in cFEC relative to the mean cFEC in the control animals of the same trial ($\overline{\text{cFECc}}$) and expressed in percentage.
- *Percentage reduction in worm burden (% wBurden reduction)*. This was computed analogously using post-mortem abomasal nematode burdens instead.

**Fig. 1:**
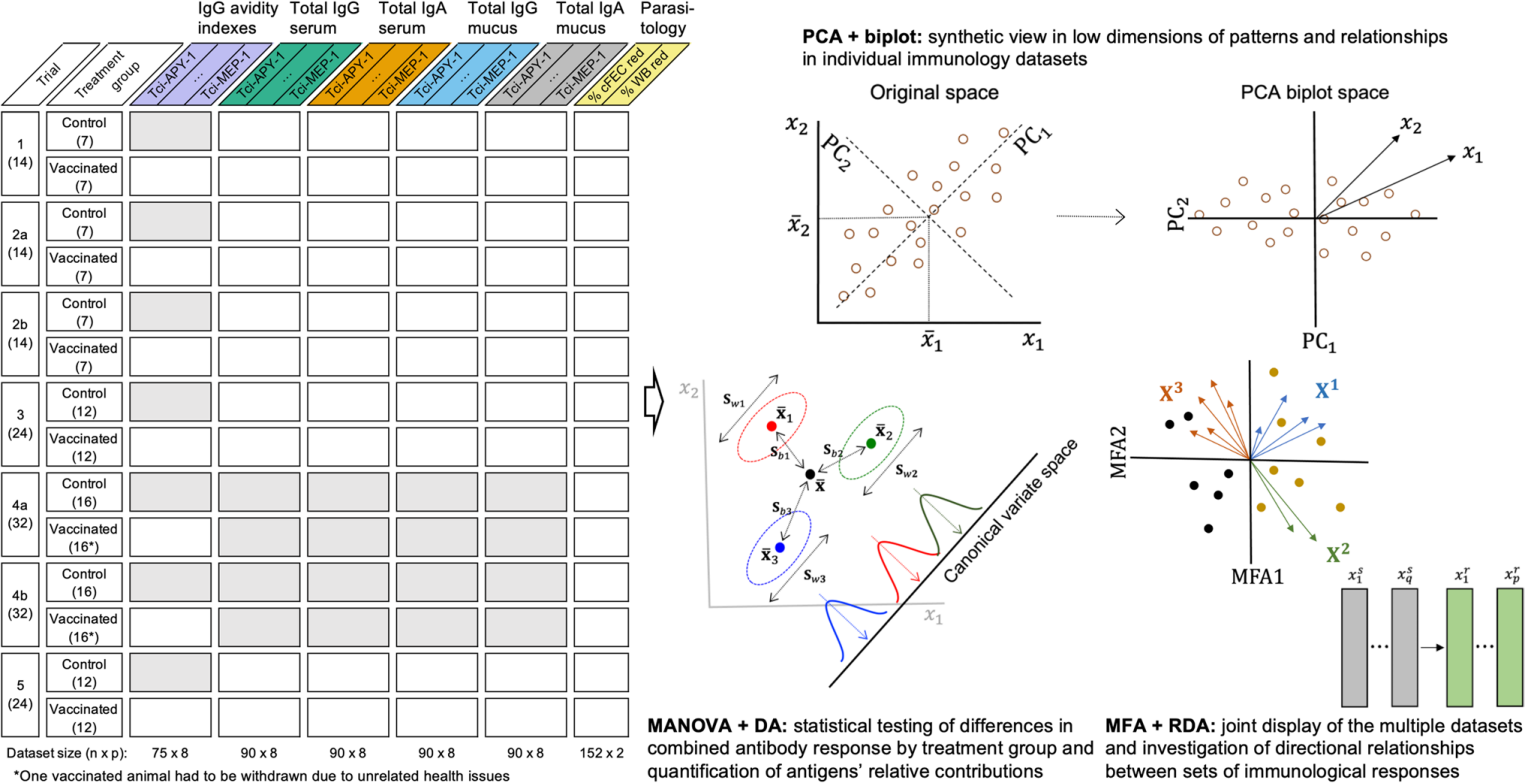
Schematic outline of datasets produced over five *T. circumcincta* vaccine trials and multivariate methods used for their analysis (see text for details). Greyed out areas indicate data not available

Figure 1 (left) shows a schematic of the data architecture, arranging the different sets of biological parameters measured in columns (distinguished by coloured headings and labels) and the successive vaccine trials and treatment groups within them in rows. The overall total number of samples was 152, however not all biological parameters were available for all samples and trials (greyed out in Fig. 1). Numbers per trials and treatment groups are indicated in parenthesis, including actual sizes of the datasets (rows $\times$ columns) at the bottom of the table. Note that one vaccinated animal had to be withdrawn due to unrelated health issues from both Trial 4a and Trial 4b.

### Multivariate data analysis methods

The following describes the basics of the selected multivariate methods and their purposes in the context of the present study. Each method or combination of them is aimed to learn from the data about a particular question of interest, and they complement each other, helping to provide an overarching view of the underlaying processes. They are not meant to be applied in any particular order, but certainly principal components analysis and the associated biplot representation as described below are typically a good starting point. These provide a synthetic view of patterns and relationships amongst samples and variables. Key summaries along with generic diagrams of the workings and main outputs are outlined in Fig. 1 (right). Some definitions and notation are established here to facilitate the exposition. Thus, a multivariate dataset consisting of $n$ samples (observations) and $p$ variables (biological parameters) is arranged as a matrix $\mathbf{X}={\left[{x}_{ij}\right]}_{n\times p}$ of elements ${x}_{ij}$ referring to the measured value for the $i$th sample in the $j$th variable, with $i=1,\dots ,n$ and $j=1,\dots ,p$ (dataset sizes $n\times p$ are detailed in Fig. 1). The centre of the data is given by the mean vector $\overline{\mathbf{x}}=({\overline{x}}_{1},\dots {, \overline{x}}_{p})$, with ${\overline{x}}_{j}$ being the ordinary arithmetic mean of the variable ${x}_{j}$. The data dispersion is summarised by the covariance matrix $\mathbf{S}={\left[{s}_{ij}\right]}_{p\times p}$, including the individual variances ${s}_{j}^{2}$ in the first diagonal and the pairwise covariances ${s}_{ij}$ off the diagonal. In practice, the correlation matrix$\mathbf{R}={\left[{r}_{ij}\right]}_{p\times p}$ with elements ${r}_{ij}={s}_{ij}/{s}_{i}{s}_{j}$ varying in $\left[\text{0,1}\right]$ is often used instead. This removes the influence of the scale of measurement and improves their comparability. Note that using $\mathbf{R}$ is equivalent to work with standardised variables ${z}_{j}=({x}_{j}-{\overline{x}}_{j})/{s}_{j}$, with mean zero and variance one, which gives all variables equal weight in the analysis. Either $\mathbf{S}$ or $\mathbf{R}$ are the main input for most multivariate statistical methods.

#### Principal component and biplot analysis

The main purpose of principal component analysis (PCA) is to reduce the dimension of a dataset ${\mathbf{X}}_{n\times p}$ by building a new dataset ${\mathbf{P}\mathbf{C}}_{n\times k}$ (with $k\le p$) of uncorrelated variables (principal components, PCs) which summarises the information in the original ones by means of linear combinations ${\text{P}\text{C}}_{i}={l}_{i1}{x}_{1}+\cdots +{l}_{ip}{x}_{p}$, with $i=1,\dots ,k$. The coefficients ${l}_{ij}$ (*loadings*), and hence the values of the ${\text{P}\text{C}}_{i}$ (*scores*), are determined so that successive PCs account for a decreasing fraction of the variation structure contained in $\mathbf{S}$ (or $\mathbf{R}$). Technically, PCA loadings and scores are commonly obtained by a so-called singular value decomposition (SVD) of the mean-centred $\mathbf{X}$ matrix (Ma and Dai 2011). Often, working with the first few $k$ PCs, those representing the main gradients of dispersion in $\mathbf{X}$, is enough to capture a notable portion of the original information, while the dimension is reduced from $p$ to $k$ for the benefit of interpretation and graphical display. Namely, the first two ($k=2$, PC_1_ and PC_2_) can be used to define a planar diagram called a biplot (Gower et al. 2010), in which both samples and variables are jointly represented by, respectively, points and rays from the origin. However, in some cases, using some subsequent PCs might be relevant to represent other underlying physical, chemical or biological processes. The biplot display is useful to explore overall variation, associations between variables, and groupings between samples. Figure 1 (right) includes a basic illustration for two variables of the move from the original space to the space spanned by the PCs. Moreover, supplementary variables can be projected onto a biplot, so that their correlations with the PCs, and hence with the variables mostly associated with them, can be explored (Graffelman and Aluja-Banet 2003). More detailed guidance on interpreting the biplot display is provided in Supplementary Information (Section S2).

PCA and biplot analysis were applied to each of the immunology datasets separately to provide an overview of patterns and relationships within them. The parasitology parameters were added to the PCA solution as supplementary variables to investigate their associations with the immunology parameters.

#### Multivariate analysis of variance and discriminant analysis

Given a dataset $\mathbf{X}$ comprising $g$ groups of samples, multivariate analysis of variance (MANOVA) tests for overall differences between mean responses by group, jointly considering all response variables and their interrelationships. The total variability ($\mathbf{T}$) of $\mathbf{X}$ is decomposed into between-groups ($\mathbf{B}$) and within-groups ($\mathbf{W}$) variability as $\mathbf{T}=\mathbf{B}+\mathbf{W}$, using the information from the data covariance structure. Thus, an important relative contribution of $\mathbf{B}$ suggests relevant differences in mean vectors. In the same spirit as PCA, MANOVA relies on optimal linear combinations of the variables called canonical variates, but here these result from maximising ${\mathbf{W}}^{-1}\mathbf{B}$ (i.e. the distinction of samples in different groups relative to samples within the same groups). Common test statistics for MANOVA make use of characteristics of the $\mathbf{B}$ and $\mathbf{W}$ matrices, such as their trace, determinant or eigenvalues, and are based on approximate $F$ distributions (Smith et al. 1962). Note that standard MANOVA relies on multivariate normality and works for regular datasets (i.e. $n>p$). The workings of MANOVA are illustrated in Fig. 1 (right) for the case of two variables and $g=3$ groups (with group means and dispersion matrices given by ${\overline{\mathbf{x}}}_{i}$ and ${\mathbf{S}}_{i}$ respectively, $i=\text{1,2},3$; and ${\mathbf{S}}_{i}$ being further decomposed into *within* (${\mathbf{S}}_{wi}$), relative to the group mean, and *between* (${\mathbf{S}}_{bi}$), relative to the overall mean, variability). The generalisation of MANOVA to include several explanatory factors or covariates defines a multivariate linear model (MLM) of the form $\mathbf{X}=\mathbf{F}\varvec{\upgamma }+\mathbf{E}$, where $\mathbf{F}$ refers to the explanatory variables, with coefficients $\varvec{\upgamma }$, and $\mathbf{E}$ is the error term (Mardia et al. 1979). Moreover, permutational MANOVA (PERMANOVA) is a semi-parametric alternative based on dissimilarity measures that uses a pseudo $F$ statistic. This works under less stringent conditions and is also applicable to wide datasets ($n<p$) (Anderson 2017). Both MANOVA and PERMANOVA assume that the variability structure is homogeneous between groups. This can be statistically tested in regular data sets using the Box’s M test (Box 1949), but note that this test is sensitive against deviation from multivariate normality. However, PERMANOVA has shown robustness to departures from such assumption in the case of balanced groups (Anderson and Walsh 2013). Otherwise, extensions of MANOVA and PERMANOVA for heterogenous dispersion and high dimensions are used (see summary of methods in Supplementary Information, Section S3). Note that canonical variates in MANOVA essentially coincide with the latent functions used in linear discriminant analysis (LDA) (Mardia et al. 1979), but the emphasis of this latter is predictive, i.e. classifying samples into groups based on a number of predictors. Quadratic discriminant analysis (QDA) is used in case of heterogeneous dispersion between groups. Predictive accuracy and relative importance of predictors can be conveniently assessed by cross-validation (Hastie et al. 2009), aiming to maximise sensitivity and specificity (Kuhn and Johnson 2013).

With the specific formulation and tests used depending on the characteristics of the data in each case (see computer codes for more details), and accounting for covariates like trial when suitable, MANOVA, MLM and PERMANOVA were applied to assess differences in combined antibody responses by treatment. Note that age was not included in this modelling as all the animals in a trial were the same age and then it would be fully confounded with trial. Moreover, DA allowed to assess the ability to distinguish groups based on antigen-specific antibody responses, quantifying the relative contributions of the different antigens.

#### Multiple factor analysis and redundancy analysis

Multiple factor analysis (MFA) aims to find a joint optimal low-dimensional representation of multiple datasets. Given $m$ multivariate datasets ${\mathbf{X}}^{1},\dots ,{\mathbf{X}}^{m}$, referring to different domains but measured for the same $n$ samples, MFA determines optimal weights for each dataset based on their variability structure. These are then applied to compute a global PCA and biplot display that allows to assess common structures and discrepancies across datasets (Abdi et al. 2013). We propose to accompany MFA with redundancy analysis (RDA), which combines multivariate regression and PCA (Legendre and Legendre 2012). Unlike PCA or MFA, RDA allows for explicit investigation of directional associations between any two datasets ${\mathbf{X}}^{r}$ and ${\mathbf{X}}^{s}$ of the collection, of say $p$ and $q$ variables respectively, the first playing the role of response dataset and the second acting as explanatory dataset. Linear combinations of the explanatory variables in ${\mathbf{X}}^{\varvec{s}}$ are sought that best explained the variation of the response matrix ${\mathbf{X}}^{r}$. PCA is then applied to these to generate a low-dimensional data representation that, unlike in standard PCA, is constrained by the explanatory dataset. Statistical significance of the RDA model is commonly tested by a global permutation test based on a pseudo $F$ statistics. Additionally, the RDA model can include a set of conditioning covariates $\mathbf{H}$, i.e. variables which potential effects are wanted to be removed to decipher the unique contribution of ${\mathbf{X}}^{s}$ to the variation in ${\mathbf{X}}^{r}$. Goodness of fit is measured by an ${R}^{2}$ statistic adjusted for the number of explanatory variables and of samples. Figure 1 (right) displays a simplified graphical illustration of the purpose and output resulting from these techniques.

MFA was used to jointly represent the antigen-specific IgG and IgA antibody response datasets along with the parasitology dataset and, thus, facilitate the identification of overall association patterns across datasets. RDA was applied to assess the potential combined influence of: (a) IgG responses in serum on IgG responses in abomasal mucus, and (b) IgA responses in abomasal mucus on IgA responses in serum.

## Results

**Fig. 2:**
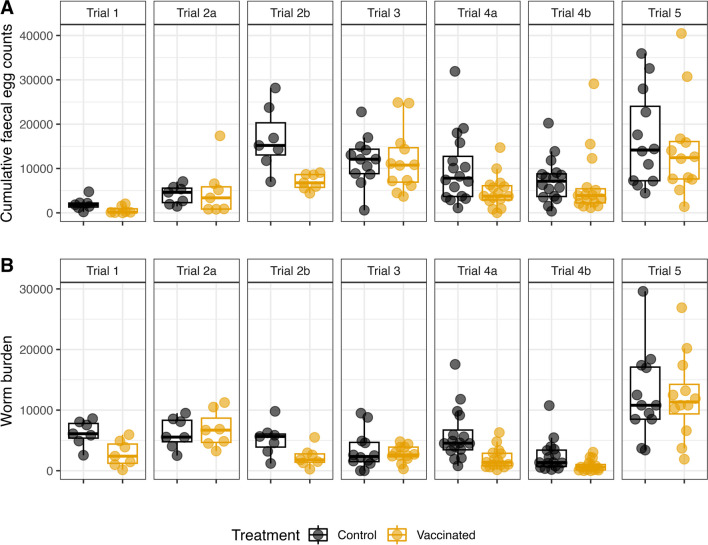
Distribution of cumulative faecal egg counts (**A**) and worm burden (**B**) by treatment group and trial. Boxplots and actual data points showed

Figure 2 depicts the observed values of the parasitology parameters cFEC and worm burden by treatment group measured across trials. The superimposed points are the actual measurements. Higher cFEC and worm burden are generally observed in the control groups. However, high variability is also notable, particularly in the later trials, where some control animals showed negligible infection levels and a few vaccinated animals for which the vaccine appears not to confer protection (“non-responders”).

### Antigen-specific IgG avidity indexes

**Fig. 3:**
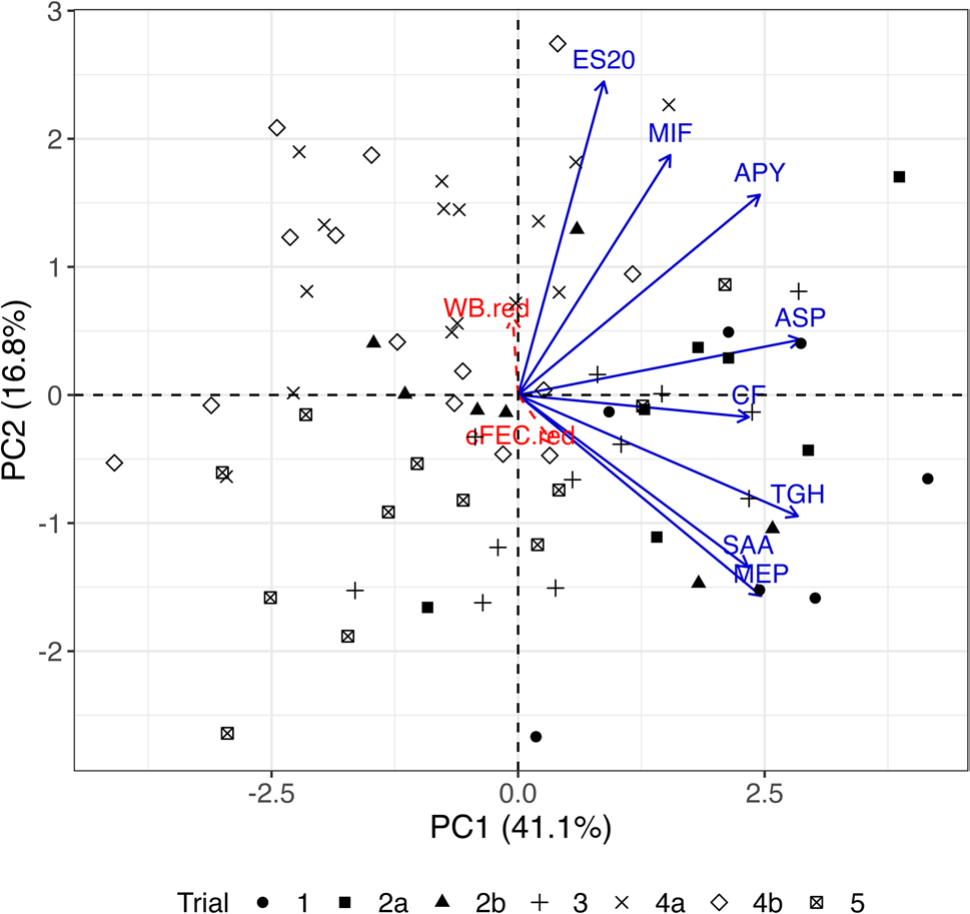
Principal component analysis biplot of antigen-specific IgG avidity indexes from vaccinated animals across vaccine trials. Red dashed-line rays indicate pathology parameters projected on the biplot as supplementary variables. Antigen ID abbreviations: APY (Tci-APY-1), ASP (Tci-ASP-1), MIF (Tci-MIF-1), TGH (Tci-TGH-2), SAA (Tci-SAA-1), CF (Tci-CF-1), ES20 (Tci-ES20) and MEP (Tci-MEP-1)

The IgG avidity indexes for the 8 antigens included in the vaccine formulation were run through PCA based on the pooled correlation matrix obtained from the vaccinated groups across trials and represented along with the samples in a 2D biplot (Fig. 3; 57.9% variability explained). The corresponding reduction in cFEC and worm burden data were added as supplementary variables. Tci-MEP-1 appeared more strongly correlated with Tci-SAA-1 and Tci-TGH-2 and weakly correlated with Tci-ES20 and Tci-MIF-1. The other antigens were evenly arranged in between these two extremes. The shortness of the rays representing the parasitology parameters indicates that the association of these with the PCA axes was poor. In fact, a linear regression fit revealed that PC1 and PC2 jointly only explained 1.81% and 1.57% of the variability in cFEC and worm burden reductions respectively (not shown). Overall, their correlations with the avidity indexes were below 0.2. Some structure is observed according to trial, although the points are fairly spread throughout. Moreover, a MLM concluded no statistically significant differences in mean avidity indexes according to sex (p = 0.1793) or weight gain (p = 0.6311), while controlling for trial effect as a blocking factor.

### Antigen-specific IgG and IgA antibody responses in serum and abomasal mucus

**Fig. 4:**
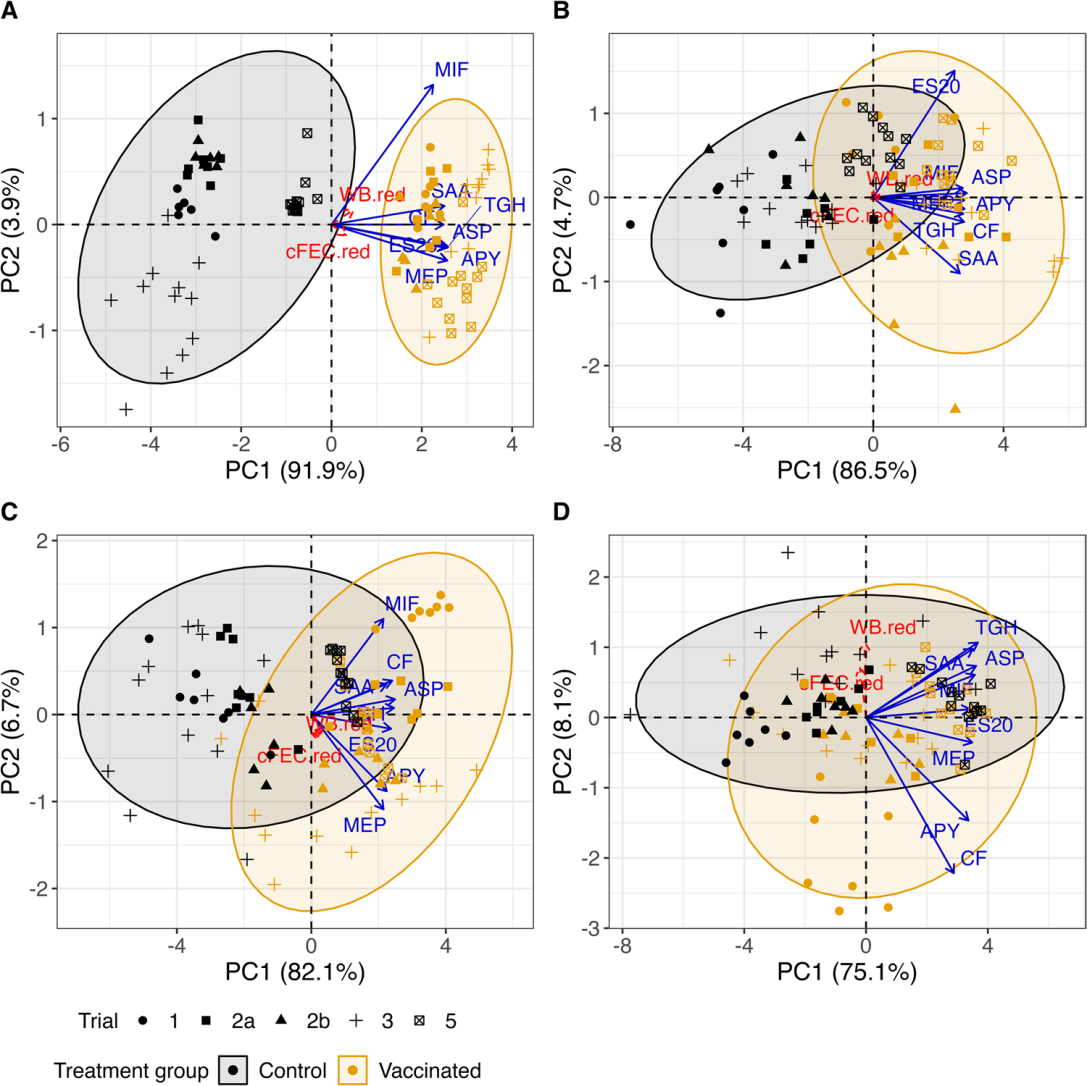
Principal component analysis biplot of antigen-specific immunological responses by treatment group: (**A**) Antigen-specific IgG in serum, (**B**) Antigen-specific IgA in serum, (**C**) Antigen-specific IgG in abomasal mucus, and (**D**) Antigen-specific IgA in abomasal mucus. Red dashed-line rays indicate pathology parameters projected on the biplot as supplementary variables. Antigen ID abbreviations: APY (Tci-APY-1), ASP (Tci-ASP-1), MIF (Tci-MIF-1), TGH (Tci-TGH-2), SAA (Tci-SAA-1), CF (Tci-CF-1), ES20 (Tci-ES20) and MEP (Tci-MEP-1)

Figure 4 shows PCA biplots by antibody location and antibody response (explained variability ranging from 83.2% for IgA in abomasal mucus to 95.8% for IgG in serum). Different symbols distinguish data by trial and 95% concentration ellipses inform about the homogeneity and direction of the group variabilities.

#### Antigen-specific IgG responses in serum

The clearest differentiation between control and vaccinated animals relates to IgG in serum (Fig. 4A; 95.8% variance explained). This is observed along the direction of the PC1 axis, with higher values for vaccinated animals in all IgG responses except for Tci-MIF-1. This latter appears closer to PC2 and determines a direction along with treatment groups are not that well distinguished: projecting all the points perpendicularly onto this ray reveals some overlapping between control and vaccinated animals, whereas the separation is complete in relation to any other antigen. The overall variability amongst the control groups is larger than in the vaccinated groups (Box’s M p < 0.0001), with this very much related to differences between trials. For example, control animals from Trial 5 form a cluster separated from the others that is closer to vaccinated animals, and Trial 3 shows the largest variability amongst control animals. As to the links with the reductions in parasitology parameters (red dashed-line rays in Fig. 4), these point towards the expected direction, i.e. increasing relative reductions in cFEC and worm burden in vaccinated animals. However, the very short length of their rays indicates that they are not well represented in the biplot and that the links with antibody responses are generally weak (1.63% and 3.97% of the variability of cFEC and worm burden reductions respectively accounted for by the two first PC axes; correlations with IgG responses of up to 0.24). Both MANOVA and PERMANOVA testing, considering homogeneous and heterogeneous dispersion matrices and controlling for trial effect, concluded statistically significant differences between treatment groups in all cases (p < 0.001). QDA provided a cross-validated predictive accuracy in classification of 99.30%, with all the antigen-specific responses contributing similarly to this separation except for Tci-MIF-1, again underlining its probable lack of suitability as a vaccine antigen (Table 1).

**Table 1:** Quadratic discriminant analysis: relative contributions of antigen-specific antibody responses in serum and abomasal mucus to distinguish between vaccinated and control animals (values between 0 and 100 from less to more contribution)

	Serum		Abomasal mucus	
QDA model predictive accuracy*	99.30%	84.40%	84.17%	80.65%
	IgG	IgA	IgG	IgA
Tci-APY-1	100	87.94	79.40	100
Tci-ASP-1	100	100	90.48	27.74
Tci-MIF-1	0	91.44	0	17.23
Tci-TGH-2	100	83.27	100	36.35
Tci-SAA-1	100	82.30	61.93	0
Tci-CF-1	95.96	80.54	70.60	64.77
Tci-ES20	100	0	91.62	19.91
Tci-MEP-1	100	90.66	92.61	63.31

*Based on 5-time repeated 10-fold cross-validation. Relative importance of predictors determined by the area under the receiver operating characteristic (ROC) curve associated with the cut-off value for the predictor that maximised sensitivity and specificity

#### Antigen-specific IgA responses in serum

The PCA biplot (Fig. 4B; 91.2% variance explained) shows a notably greater overlapping between groups than in the IgG case. All antigen-specific IgA responses appear highly correlated along the PC1 axis, except for Tci-SAA-1 and, specially, Tci-ES20 which yielded fairly comparable values for control and vaccinated animals. The overall dispersion was somewhat larger amongst the vaccinated animals (Box’s M p < 0.0001). MANOVA and PERMANOVA tests concluded statistically significant differences between treatment groups after accounting for trial effect (p < 0.001). Distinguishing the points by trial reveals that the smallest separation occurred in Trial 5 as seen for IgG. The parasitology parameters appear very poorly represented (0.09% and 0.66% variability explained for cFEC and worm burden reductions respectively; correlations with antibody responses < 0.15). QDA provided a cross-validated predictive accuracy of 84.40% when antigen-specific IgA responses in serum were used, with Tci-ASP-1 and Tci-ES20 being the ones contributing the most and the least respectively (Table 1).

#### Antigen-specific IgG responses in abomasal mucus

The PCA biplot (Fig. 4C; 88.8% variance explained) shows separation between control and vaccinated animals along the PC1 axis, although there is high variability and some overlapping. Particularly, control and vaccinated animals from Trial 5 are the least separated, and there are also several vaccinated animals in Trial 3 that had responses comparable to control animals. In contrast, the treatment groups in Trial 1 are the most far apart from each other. Referring to the parasitological parameters (Fig. 2A and B), Trials 3 and 5 show the least differences between vaccinated and control groups, indicating that serum and mucosal IgG distinctions between vaccinated and control groups may be indicative of the level of vaccine efficacy in trials. The correlations between antigen-specific responses are not as homogeneous as seen in the previous cases, with Tci-MIF-1 as before but also Tci-MEP-1 and Tci-APY-1 splitting apart from the main group. The highest correlation is observed between Tci-CF-1 and Tci-SAA-1, which is compatible with the results above. The overall dispersion was significantly different between treatment groups (Box’s M p < 0.0001) and both MANOVA and PERMANOVA tests concluded statistically significant differences between groups after accounting for trial effect (p < 0.001). In line with previous results, the parasitology parameters appear poorly represented (3.28% and 3.27% variability explained for cFEC and worm burden reductions respectively; correlations with antibody responses < 0.19). QDA provided a cross-validated predictive accuracy in classification of 84.17%, with Tci-TGH-2 and Tci-MIF-1 contributing the most and least respectively (Table 1).

#### Antigen-specific IgA responses in abomasal mucus

The PCA biplot for this case (Fig. 4D; 83.2% variance explained) shows the largest overlap between vaccinated and control animals. In line with previous results, responses in Trial 5 are hardly distinguishable between groups, but here this is also the case for others such as Trial 2. Unlike previously, groups in Trial 1 are not that neatly separated here. Vaccinated animals appear markedly disperse, with the distinction between groups is mostly due to Tci-CF-1 and Tci-APY-1. The correlations between IgA responses are more varied than before and patterns previously observed are not evident in this case. The overall dispersion was again significantly different between groups (Box’s M p < 0.0001) and MANOVA and PERMANOVA tests concluded statistically significant differences in the combined mean antibody responses after accounting for trial effect (p < 0.002). The parasitology parameter measurements clearly align with the PC2 axis and appear disconnected from the antibody responses (1.16% and 3.94% variability explained for cFEC and worm burden reductions respectively; correlations with antibody responses < 0.17). QDA provided a cross-validated predictive accuracy of 80.65% when allocating samples to groups, with Tci-APY-1 and Tci-SAA-1 contributing the most and least respectively (Table 1).

### Relationships between antibody levels in serum and abomasal mucus

**Fig. 5:**
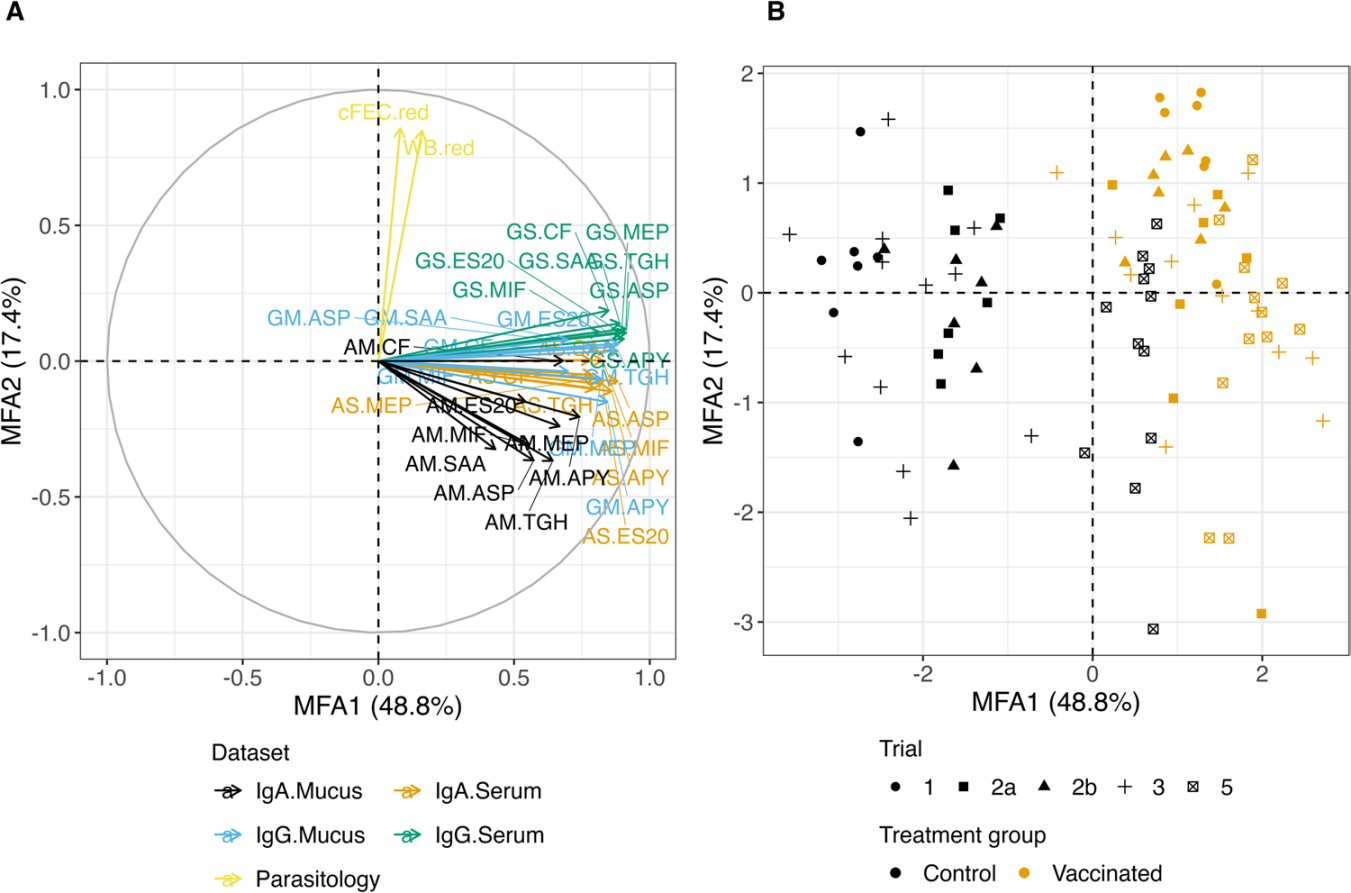
Multiple factor analysis biplot of immunology and parasitology datasets: (**A**) variables plot and (**B**) samples plot. Letters preceding antigen name refer to either IgG or IgA responses (G or A) and to either serum or abomasal mucus (S or M). Antigen ID abbreviations: APY (Tci-APY-1), ASP (Tci-ASP-1), MIF (Tci-MIF-1), TGH (Tci-TGH-2), SAA (Tci-SAA-1), CF (Tci-CF-1), ES20 (Tci-ES20) and MEP (Tci-MEP-1)

MFA was applied to the four antibody response datasets along with the parasitology parameters. Figure 5 shows the resulting consensus biplot based on the first two MFA axes (labelled MFA1 and MFA2; 66.2% variance explained). It is split into variables plot (Fig. 5A; rays coloured by dataset) and samples plot (Fig. 5B; points distinguished by treatment group and trial) to facilitate visualisation. The first MFA axis essentially characterises the contrast between control and vaccinated animals, with overall higher antibody responses in vaccinated animals. The second MFA axis mostly accounts for the relative reduction in the parasitology parameters. It can be observed that IgA levels in serum are in general more correlated with IgA responses in abomasal mucus than with any other group of parameters (AM and AS rays closer to each other below the horizontal reference line). The same relationship applies to IgG responses (GS and GM rays closer to each other above the horizontal reference line). Antibody responses appear poorly associated with the measures of relative reduction in cFEC and worm burden used. Measurements across trials were generally consistent except for Trial 5, which shows the poorest distinction between control and vaccinated animals.

RDA allowed to investigate to what extent antigen-specific IgG response in serum might explain IgG response in abomasal mucus. Treatment group and trial effects were added to the model as conditioning covariates to determine the fraction of the variation of the IgG response in abomasal mucus uniquely explained by IgG response in serum. The RDA model was statistically significant (p = 0.001) and had adjusted ${R}^{2}$ equal to 7.88%, with the total variation in antigen-specific IgG responses in abomasal mucus broken down as: 58.65% explained by the conditioning terms, 10.66% uniquely explained by the IgG responses in serum, and the remaining 30.69% being residual variation. RDA was again used to investigate the potential influence of antigen-specific IgA response in abomasal mucus on IgA response in serum, including group and trial as conditioning variables. No statistically significant association was concluded (p = 0.157; adjusted ${R}^{2}$ = 0.92%). In particular, the observed variability in IgA response in serum was decomposed into 69.93% explained by the conditioning terms, 3.65% explained by IgA response in abomasal mucus, and 26.41% residual variance.

## Discussion

A comprehensive assessment of the ability of a candidate vaccine to induce immune responses that can effectively protect from infection and disease is fundamental to both understanding the action of the vaccine but also to optimise the prototype. Particularly, the aim of most vaccines against parasitic gastrointestinal nematodes is to prevent contamination of the pasture with parasite eggs and thus reduce the infection pressure for the current and future groups of livestock grazing that pasture (Nisbet et al. 2019). The results from the current study identified meaningful overall differences between vaccinated and control groups in relation to the fundamental parasitology and immunology parameters collected over a series of vaccine trials against the *T. circumcincta* nematode.

 Information about the antigens most highly associated with vaccine efficacy is valuable, particularly when attempting to reduce the number of antigens in complex vaccine formulations which is essential in bringing reliable, effective, inexpensive vaccines to commercial reality. The results suggests that the association of the parasitology parameters with the antigen-specific IgG avidity indexes was generally poor, although relative reduction in cFEC appeared mostly linked to Tci-MEP-1 and reduction in worm burden appeared mostly linked to Tci-ES20 (Antigen-specific IgG avidity indexes section). When looking at antigen-specific antibody responses, the data analysis revealed that the largest differences between treatment groups related to IgG response in serum, with Tci-MIF-1 appearing to be a poor immunogen even in the presence of the adjuvant Quil A, which drives a strong antibody response with many antigens (Antigen-specific IgG and IgA antibody responses in serum and abomasal mucus section). As the vast majority of registered commercial vaccines work by driving a high circulating IgG response, this information is valuable in eliminating Tci-MIF-1 from further vaccine development. To some lesser extent, mucosal IgG may be indicative of vaccine efficacy as well and again suggested poorer differentiation based on Tci-MIF-1. Moreover, antigen-specific IgG responses (serum and mucosal) against Tci-MEP-1 and Tci-APY-1 may be more likely to be associated with vaccine efficacy in reducing cFEC and these antigens together or separately may be considered in a simplified version of the vaccine.

The variability in antibody responses was generally higher amongst control animals, showing also patterns according to trial. Thus, control animals from Trial 5 displayed particularly high IgG antibody levels in serum, which may be explained by their very high worm burdens in this trial compared with other trials (see Fig. 2B) where greater exposure of the host to high native antigen levels from this high parasite load may be driving the enhanced immune reaction to native versions of the vaccine antigens being secreted by the worms.

The differentiation between treatment groups based on IgA antibody responses was significant although less evident than in the IgG case, particularly in abomasal mucus, with the parasitology parameters appearing to be very poorly associated with them. This suggests that measurement of antigen-specific serum IgA levels is not likely to be useful in refining or simplifying the vaccine further.

The combined application of the MFA and RDA methods allowed to summarise the evidence across the multiple biological parameter datasets to provide an integral view of the efficacy of the prototype vaccine, gaining insight into the overall associations between antibody levels in serum and abomasal mucus (Relationships between antibody levels in serum and abomasal mucus section). Mucosal IgG in ruminants is generally not produced locally in the gut and hence IgG present on the mucosal surface of the abomasum is more likely to be derived from cross-membrane transport of circulating IgG into gut secretions (McNeilly et al. 2007). The subclass IgG1 is prominent within mucosal secretions of sheep and cattle and it is thought to play an important role in mucosal immunity (Butler 1983). There is also evidence that IgG1 is actively transported from serum to the mucosal surface (Newby and Bourne 1976), potentially via neonatal Fc receptor (Mayer et al. 2002). The results suggest that most variation in antigen-specific IgG responses in abomasal mucus was related to treatment group and trial effects and about 10.66% was uniquely explained by IgG responses in serum. In contrast, antigen-specific IgA is locally produced in abomasum-associated lymphoid follicles and is assumed to shuttle across the gut by transcytosis through epithelial cells via the polymeric IgA receptor (Gutzeit et al. 2014) with no specific mechanism for transfer of this antibody subclass into serum. This was supported by the RDA results, which identified a non-significant 3.65% of the variability in IgA response in serum as being uniquely explained by IgA response in abomasal mucus. Note however that these results might be affected by inter-individual variation in the amount of mucosal secretions collected at post-mortem that could not be accounted for due to lack of records. Furthermore, relationships between serum and mucosal antibody levels may have been compromised due to the time-lag between serum and mucosal antibody measurements.

One obvious caveat to the analysis presented in this study is that vaccine efficacy may be influenced by other immunological parameters such as cell-mediated immunity. Indeed, a recent transcriptomic analysis of the abomasal mucosa from sheep immunised with the same prototype *T. circumcincta* vaccine identified early T-helper type-1 immune responses were correlated with protection (Liu et al. 2022), suggesting that cellular immune responses may also be useful to explore using the analysis framework presented in this study.

The current feasibility to collect increasingly complex multi-variable datasets demands an increased sophistication of the data analysis and statistical modelling that is beyond the basic tests and univariate analyses traditionally conducted in the context of animal vaccine efficacy trials. The use of multivariate statistical techniques as demonstrated in this work considers the several variables simultaneously, providing an overall view that accounts for their possible inter-dependence. They provide a solid quantitative basis to advance the understanding of pathogens and how they interact with their host organisms and are ideally placed to underpin current trends such as systems immunology (Fong et al. 2018), where a wider view across transcriptomic, proteomic, metabolomic, cellular assays, etc. is promoted to identify biomarkers of protection.

In any case, it is important to stress that the availability of sophisticated analytical methods and associated computational tools should not displace the paramount importance of, firstly, having well-defined biological questions and meaningful data to tackle them; and, secondly, having a sufficient understanding of variation and uncertainty along with a critical knowledge of the functioning, key technical assumptions, and scope of application. The multivariate methods discussed in this work, as for many other statistical methods, assume variables measured at a continuous level and linear structure underlaying the data. Related to this, they are also better behaved with symmetric (normal) data distributions, particularly when inferential analysis is involved. In many applications linearity is a good enough approximation to capture main patterns. Moreover, some preliminary data transformation can often help to achieve it and to symmetrise the data. Thus, log-transformation on right-skewed immunology parameters contributed to downplay the issue here.

Where these assumptions are clearly unrealistic, or also to address dimensionality reduction with not only continuous features, some non-linear counterparts have been proposed (Makarenkov and Legendre 2002; Lee and Verleysen 2007). These extensions are however not so readily available in software packages for general practitioners, and they usually involve a higher level of technical expertise to be effectively used and interpreted. Amongst the methods examined, MANOVA and LDA are those subject to the most stringent assumptions given their parametric inferential nature. Limitations and alternatives like PERMANOVA or QDA for the case in which basic assumptions are not met were briefly discussed in “Multivariate data analysis methods” section. As illustrated in “Results” section, the easy access to software routines also allows to apply different methods and approaches in combination when sensible and convenient to have a more solid view of the information contained in the data.

Furthermore, there exist alternative multivariate approaches and extensions that might be convenient depending on the characteristics of the data. For example, there is an extensive statistical literature regarding the treatment of missing data in multivariate data sets, often involving a preliminary sensible imputation of the missing cells (as we applied to the avidity data set in this study) (Molenberghs et al. 2014; van Buuren 2018), as well as regarding the identification and treatment of multivariate outliers (Rousseeuw and Van Den Bossche 2018). Moreover, versions of PCA have been developed to deal with missing data within the own fitting process (Josse and Husson 2016) or to downplay the influence of potential outliers in the results (Candes et al. 2009). There are also “sparse” versions of PCA (Zou et al. 2006) and redundancy analysis (Csala et al. 2017) which facilitate interpretation and identification of biomarkers in highly dimensional data. Regularization methods are another area of intense study for regression and classification modelling in this context. They simultaneously deal with model estimation and variable selection by applying penalty terms to identify the most relevant variables. Modern methods for integrative analysis of multiple datasets of varied nature and dimensionality, e.g. multi-omics data, make use of these approaches (Meng et al. 2016).

In conclusion, the novel application of a selection of multivariate methods contributed to establish the efficacy of a recent recombinant protein eight-antigen prototype vaccine against teladorsagiasis, a major disease in sheep worldwide. Beyond what is achievable by ordinary statistical protocols routinely used in animal vaccine efficacy trials, the multivariate approach provided valuable biological insight into the main drivers of immunological protection and their interactions; helping to identify antigens most closely associated with improved parasitology outcomes and indicating directions for further optimisation of the vaccine formulation. The results also suggest that antibody avidity and levels of antigen-specific antibody appear not to be sufficient by themselves to explain vaccine efficacy, or inefficacy, and that further predictors of vaccine-induced immunity should be sought. The approach discussed here could be analogously applied to similar studies in other research contexts to assess the efficacy and guide the optimisation of prototype vaccines. Or, in fact, to any study where multiple biological parameters are measured and decision making could be enriched and streamlined by this form of joint analysis. The methods discussed here could be applied in similar studies to assess the efficacy and guide the optimisation of prototype vaccines. In the spirit of Open Science, the data and the computer codes are made freely available to facilitate use by practitioners.

### Supplementary information

Below is the link to the electronic supplementary material.

ESM 1 (PDF 108 KB)

## Data Availability

The data and computer scripts used, written for the R open system for statistical computing (R Core Team 2022), are freely available on Github (https://github.com/Japal/MDA-vaccine-trials).
