# Supplementary material for: A curated multivariate approach to study efficacy and optimisation of a prototype vaccine against teladorsagiasis in sheep
Source: Vet Res Commun. 2023 Sep 14;48(1):367–79. doi: 10.1007/s11259-023-10208-9 (PMC10810991; doi:10.1007/s11259-023-10208-9)
Supplement: Supplementary file 1 — (PDF 108 KB) [file 11259_2023_10208_MOESM1_ESM.pdf]

Supplementary information file

A curated multivariate approach to study efficacy and optimisation of a prototype vaccine against teladorsagiasis in sheep

Veterinary Research Communications

Javier Palarea-Albaladejo[1,2], Tom N. McNeilly[3], Alasdair J. Nisbet[3]

[1]Department of Computer Science, Applied Mathematics and Statistics, University of Girona, Girona, Spain
[2]Biomathematics and Statistics Scotland, JCMB, The King's Buildings, Peter Guthrie Tait Road, Edinburgh, Scotland, UK
[3]Moredun Research Institute, Pentlands Science Park, Bush Loan, Penicuik, Scotland, UK

Corresponding e-mail address: javier.palarea@udg.edu

S1. General immunisation and sampling regime of the vaccine trials

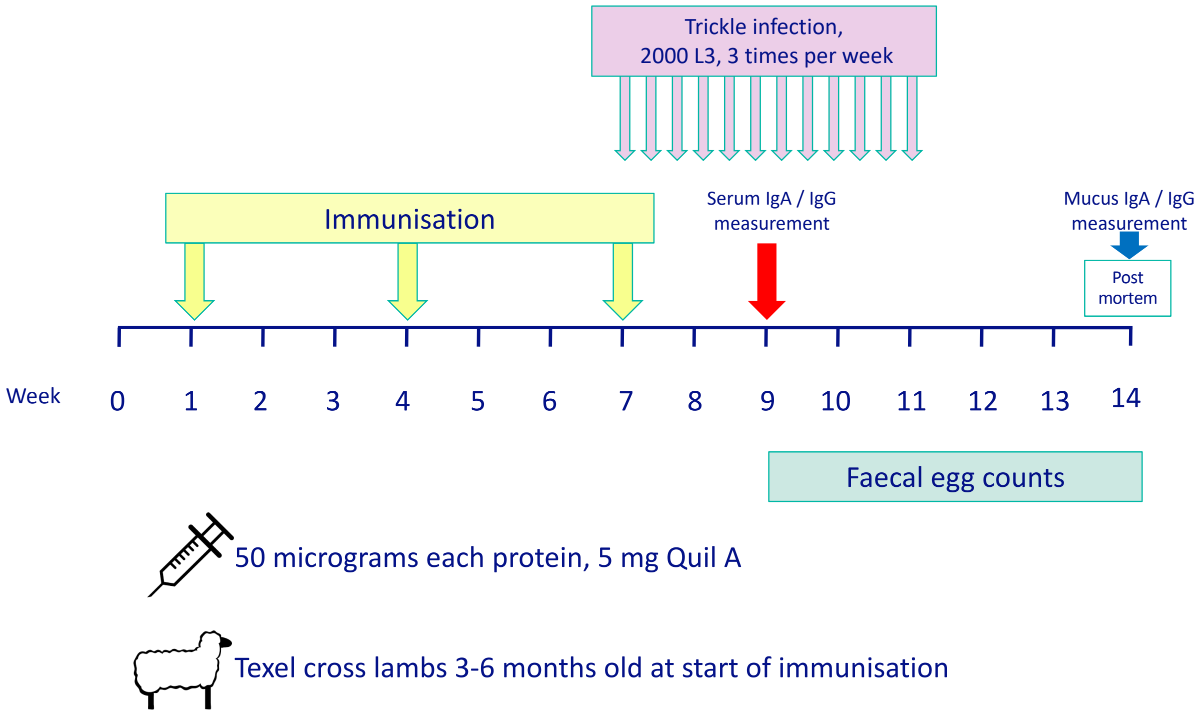

S2. Guidance on interpreting PCA biplots with supplementary variables

Biplots are meant to summarise the main features of a multivariate data set in low dimensions. They can be understood as a generalisation of scatterplots that includes a number of non-orthogonal axes (represented by rays in the biplot) referring to the several variables. The values of each sample or observation (represented by a points in the biplot) on these variables can be approximately recovered by perpendicularly projecting the points onto these axes. This allows to determine the relative position of the samples in relation to the different variables.

The distance between points approximates the similarity between samples in terms of the original variables. The coordinates of the rays indicate directions of increasing values on the variables, with the origin of the coordinate system representing the mean vector $\bar{\mathbf{x}}$ of the original dataset. The angles (the proximity) between rays approximate the correlations between the variables they represent, with 0°, 90° and 180° angles corresponding with no correlation (independence), perfect positive correlation, and perfect negative correlation respectively for reference. Thus, for instance the closer two rays are in the biplot the higher the positive correlation between the associated variables. The length of the rays approximates the standard deviation of the variables when non-standardized variables are used as input (covariance-based biplot). When the variables are standardized to have unit variance (correlation-based biplot; as used in this work to homogenise variability across variables), then the length of the rays indicates how well the variables are represented in the biplot, with relatively short rays indicating poorer representation. The angles of the rays with the vertical and horizontal reference PC axes crossing at (0, 0) indicate how each variable associates with each PC, with again the shorter the distance the stronger association with a PC.
Finally, it is of interest on some occasions to represent in the biplot variables that were not included in the original PCA (i.e. they did not participate in the determination of the PCs) because they represent an entirely different kind of information. However, including them as supplementary variables is useful to investigate how they relate to the first ones. Thus, they are displayed by additional rays on the space spanned by the PCA biplot (differentiated from the rays of the original variables) which coordinates are related to their correlation with the PC axes and, hence, to their correlation with the variables most associated to them. Thus, this representation allows to link original variables, supplementary variables and samples. The length and orientation of the supplementary variables rays indicate the strength and direction of those relationships, with proximity between rays being related to correlation as described above. In our case, the immunology datasets were used in PCA and then their relationships with the parasitology parameters were investigated by adding these latter as supplementary variables.

S3. MANOVA type comparisons in the case of heterogeneous dispersion and high-dimensional data

Heterogeneity of matrices de covariances is an old problem in multivariate statistical inference about mean differences between groups. There have been several proposals in the literature and it is currently an active area of research. Some methods include for example the James' test (James, 1954), parametric and nonparametric bootstrap-based methods that allow for minimal assumptions on the data (Konietschke et al. 2015; Bathke et al. 2018), a modified ANOVA-type statistic (MATS) for factorial designs (Friedrich and Pauly, 2018), nonparametric rank-based approaches that work with extended classes of data like ordinal data (Dobler et al. 2020) and a modification to the ordinary PERMANOVA test statistic for the heterogeneous case (Anderson et al. 2017). Moreover, high-dimensional data including more variables than samples, nowadays routinely generated in e.g. molecular biology research, often challenge ordinary MANOVA methods for regular multivariate data. Recent works extending MANOVA for this case include the sparse method of Cai and Xia (2014) for the case of multiple groups and the method introduced in Kong and Harrar (2021) that relaxes the condition of weak dependence between variables commonly required by othert proposals.

References

Anderson MJ, Walsh DCI, Robert Clarke K, Gorley RN, Guerra-Castro E (2017) Some solutions to the multivariate Behrens–Fisher problem for dissimilarity-based analyses. Aust N Z J Stat 59: 57-79. https://doi.org/10.1111/anzs.12176

Bathke AC, Friedrich S, Pauly M, Konietschke F, Staffen W, Strobl N, Höller Y (2018) Testing mean differences among groups: multivariate and repeated measures analysis with minimal sssumptions. Multivariate Behav Res 53: 348-359. https://doi.org/10.1080/00273171.2018.1446320

Cai TT, Xia Y (2014) High-dimensional sparse MANOVA. J Multivar Anal 131: 174-196. https://doi.org/10.1016/j.jmva.2014.07.002

Dobler D, Friedrich S, Pauly M (2020) Nonparametric MANOVA in meaningful effects. Ann Inst Stat Math 72: 997-1022. https://doi.org/10.1007/s10463-019-00717-3

Friedrich S, Pauly M (2018) MATS: Inference for potentially singular and heteroscedastic MANOVA. J Multivar Anal: 165: 166-179. https://doi.org/10.1016/j.jmva.2017.12.008

James GS (1954) Tests of linear hypotheses in univariate and multivariate analysis when the ratios of the population variances are unknown. Biometrika 41: 19-43.

Kong X., Harrar SW (2021) High-dimensional MANOVA under weak conditions. Statistics 55: 321-349. https://doi.org/10.1080/02331888.2021.1918693

Konietschke F, Bathke AC, Harrar SW, Pauly M (2015) Parametric and nonparametric bootstrap methods for general MANOVA. J Multivar Anal 140: 291-301. https://doi.org/10.1016/j.jmva.2015.05.001
